# Fostering Solidarity Among Ethnic Minority Groups: Addressing the Role of Inter-Minority Contact in Cross-Cultural Contexts

**DOI:** 10.5334/irsp.1096

**Published:** 2025-10-13

**Authors:** Giulia Rosa Policardo, Francesca Prati, Cayir Burak, Jasper Van Assche, Monica Rubini

**Affiliations:** 1 University of Florence, IT; 2 University of Bologna, IT; 3 Libre University de Bruxelles, BE

**Keywords:** intergroup contact, solidarity, inter-minority, disadvantaged group, collective action

## Abstract

The present research investigates when and how contact among ethnic minority groups members is linked with solidarity, as a joint effort aimed at mitigating social inequalities. Two cross-sectional studies in Belgium (Study 1a) and Turkey (Study 1b) with North African immigrants and Iraqi immigrants respectively, were conducted. Convergent results supported the assumptions that having positive inter-minority contact is linked with solidarity with other ethnic minorities and the association is mediated by affective injustice. In addition, across both studies, having positive inter-minority contact attenuated the link between positive contact with the majority group and lower inter-minority solidarity and it enhanced the link between negative contact with the majority group and higher inter-minority solidarity. Inconsistent results were found for negative inter-minority contact. Only in the Turkish context, it was positively associated with affective injustice that in turn showed an indirect effect in the link between negative inter-minority contact and solidarity. Overall, results underscore the pivotal role of positive inter-minority contact in fostering cooperation among ethnic minority groups and highlight for the first time the complex interplay between the valence of majority-minority and inter-minority contact experiences.

According to the European Network Against Racism (Sanaullah 2023), around 10 percent of people living in Europe, corresponding to roughly 50 million, have ethnic minority backgrounds.

Data from the Pew Research Center (Wike, Stokes & Simmons 2016) suggests that this trend of cultural diversity and foreign-born population has steadily increased in Europe and across the Western world in the last decades, drawing further attention to issues such as inter-ethnic tolerance and equality. Despite this emerging trend of increasingly multicultural and multi-ethnic societies, social psychological research on intergroup relations has focused almost exclusively on attitudes and interactions between members of majority and minority groups. Similarly, collective action research has also traditionally focused on minority or majority group members’ motivations and actions to support the lower status group (van Zomeren et al. 2008), rather than assessing solidarity among disadvantaged groups. The only exception is represented by few studies on inter-minority contact (Cernat 2019; Dixon et al. 2010; Visintin et al. 2017) showing that positive contact among ethnic minorities is associated with higher support for pro-outgroup minority policies, while negative contact reveals an opposite pattern (see Policardo, Karataş & Prati 2025 for a review). To further understand when and how inter-minority contact can promote or inhibit alliances among ethnic minorities, we investigated positive and negative inter-minority as well as majority-minority contact. While previous research addressed the mediating role of emotions in inter-minority contact (Visintin et al. 2017), this research addresses for the first time affective injustice (Van Zomeren et al. 2008), as it might contribute to explain how contact between ethnic minority groups can mobilize one’s group in support of another one. In order to secure more generalizable evidence, we carried out our studies with two ethnic minority groups (i.e., North-African migrants in Belgium; Iraqi migrants in Turkey) living in more and less inclusive countries in terms of integration and immigration policies (see Migrant Integration Policy Index, MIPEX, 2020; https://www.mipex.eu/).

## Intergroup Contact Valence From the Perspective of Ethnic Minority Groups

A large body of research since the original proposition of intergroup contact theory (Allport 1954) has supported the relation between intergroup contact and reduced outgroup prejudice. The meta-analytic findings of Pettigrew and Tropp (2006) provided strong evidence that positive majority-minority contact typically improves attitudes toward outgroups, consistently among members of both groups (Allport 1954). However, negative compared to positive intergroup experiences might be more frequent for disadvantaged group members, increasing their anxiety about future intergroup contact (Tropp 2003) that in turn might prevent them from having positive outgroup attitudes. Research has illustrated the distinct roles of negative and positive intergroup contact of minority group members on their willingness to engage in collective action aimed at decreasing societal inequalities faced by their own group (Graf, Paolini & Rubin 2014). While positive, especially intimate, contact with the majority group can lead to a reduction of minority group members’ collective action intentions (Hässler et al. 2020; Saguy et al. 2009), negative intergroup contact is more likely to facilitate minority group members’ support for social change and shows stronger effects compared to positive contact (see Reimer & Sengupta 2023 for a meta-analysis).

Up to now, research on intergroup contact and social change suggests that other intervening factors can affect this relationship, and further research is needed to disentangle underlying processes that enhance or inhibit minority members’ willingness to engage in different activities to reduce intergroup inequalities (Di Bernardo et al. 2021). Among the different factors that can facilitate minority groups’ engagement to promote social change, one may find inter-minority contact (Hindriks, Verkuyten & Coenders, 2014; Visintin et al. 2017), such as the quantity and quality of contact between members of different minority or disadvantaged groups. In this regard, a small but consistent corpus of research, across a variety of contexts and groups, showed a positive link between inter-minority contact and positive attitudes toward ethnic minority groups, using a multiplicity of measures (Bowman & Griffin 2012; Hindriks, Verkuyten & Coenders 2014; Tropp et al. 2018; Van Laar et al. 2005). Yet, the development of positive intergroup attitudes does not necessarily imply solidarity and support for collective action in favor of other minorities’ rights.

## Inter-Minority Contact and Support for Social Change

The investigation of the relationship between inter-minority contact, and its role on either facilitating or hindering support for other minority groups, has been addressed by very few studies so far (see Policardo, Karataş & Prati 2025 for a systematic review). Among them, a cross-sectional study addressed the influence of inter-minority contact of the Indian minority in post-apartheid South Africa on political support toward their Black neighbors (Dixon et al. 2015). Results showed that contact with Blacks engendered a sense of political support among Indians, strengthening the shared political objectives and actions between the two disadvantaged groups to support social change. This association was partially explained by Indians’ awareness of discrimination faced by the ethnic minority outgroup, suggesting that inter-minority contact that enables cultural exchanges can foster political support to challenge the status quo. Going beyond this evidence, the distinct role of positive and negative inter-minority contact was addressed in a cross-sectional study considering the perspective of the Turkish ethnic minority toward the lower status group of Roma people living in Bulgaria (Visintin et al. 2017). While positive inter-minority contact was associated with more support for pro-Roma policies, negative contact was related to less support for those policies and intergroup emotions mediated those associations. Negative contact was related to negative emotions such as feelings of anxiety, contempt, and anger and negatively associated with positive emotions such as empathy and trust. Yet other psychological processes linked to intergroup contact and collective action could contribute to explain their association. In this regard, among different motivational factors to collective action (i.e., identity, efficacy, and morality) the strongest role is played by politicized identification and the affective experience of injustice (Agostini & Van Zomeren 2021; Van Zomeren et al. 2008). In particular, injustice can be linked to inter-minority contact, since research showed that during social interactions, ethnic minority members are more comfortable in talking about social inequalities with members of their ingroup or other minority members (Hindriks, Verkuyten & Coenders, 2014; Sanchez, Kalkstein & Walton 2022) sharing similar discriminations compared to the majority group. Among different measures of injustice, such as perceptions and feelings of fairness, the affective one, encompassing a constellation of moral emotions such as anger, indignation and dissatisfaction toward unfair social conditions (Van Zomeren et al. 2008), was proven to be the strongest predictor of collective action. Thus, affective injustice may contribute to explain inter-minority engagement in support for social change.

A more recent study (Cernat 2019) has addressed the influence of ethnic minorities status and their relationship with the majority group on reciprocal support for social change. Close inter-minority contact of the higher status minority of Hungarians in Romania was related to increased support for general social policies but not for those in favor of the relatively lower status minority group of Roma. On the contrary, close inter-minority contact of Roma with Hungarians was positively related to support for both general and specific policies in favor of the relatively higher status ethnic minority (Cernat 2019). The beneficial role of inter-minority contact on support for social change was restricted to higher status minority members. Even more, closer minority members’ interactions with the majority (i.e., cross-group friendship) were associated with reduced political support for policies that would be beneficial for their own and the other marginalized minority group (Cernat 2019). In this vein, friendship with the majority group may interfere with establishing minority coalitions capable of challenging existing inequalities and injustices within the host society (Craig & Richeson 2012). Indeed, given that inter-minority solidarity aims to reducing the high privileges of the majority thus challenging the status quo, one’s minority group ties with another minority, as well as the quality of relations with the majority group influence the engagement in actions aimed to social change. To date, to our knowledge, no other research has yet examined the interplay of inter-minority contact and contact with a majority group. Moreover, given the distinct but often understudied role of negative contact (Schäfer et al. 2021), further research is needed to assess the separate role of both positive and negative experiences with the majority group and the minority outgroup in building inter-minority coalitions to challenge the status quo.

## Intergroup Solidarity

The literature on intergroup solidarity does not report a clear-cut definition of intergroup solidarity (e.g., Abdelhadi & O’Brien 2020; Louis et al. 2019; Selvanathan, Lickel & Dasgupta, 2020; Subašić, Reynolds & Turner 2008). However, one of the most recent contributions on this debate has conceptualized solidarity as a ‘form of advocacy with marginalized groups enacted by members of other groups on the social issue in question, in order to promote social change by challenging the system of discrimination in place’ (Selvanathan, Lickel & Dasgupta 2020). It can be exclusively in favor of an outgroup, or it can derive from a shared interest between one’s group and other groups. When different groups act in solidarity, they do so in a way that capitalizes on sub-group differences—in membership composition, position in the social structure, or access to resources—in order to achieve the common purpose of support for social change. Nevertheless, groups status matter, because even though majority-minority (i.e., advantaged-disadvantaged) groups’ solidarity is encouraged to achieve long-term social change (Subašić, Reynolds & Turner 2008), these groups’ solidarity answers to different needs (i.e., advantaged group members’ need for moral acceptance and disadvantaged group members’ need for respect), influencing the dynamics and forms of this (not necessarily positive) alliance (see Selvanathan, Lickel & Dasgupta 2020 for a review). Indeed, solidarity encompasses multiple dimensions, and can be studied at the emotional, attitudinal and behavioral levels. While central cognitive processes underlying intergroup solidarity are the perception of a shared or superordinate identity (Gaertner & Dovidio 2014) and the recognition of interdependence or ‘linked fate’ (Craig & Richeson 2012), at the emotional level, empathy and moral emotions (i.e., moral outrage and collective guilt) play pivotal roles to catalyze group participation or motivate majority group members’ reparative and supportive actions (Branscombe, Slugoksi & Kappen 2004). Moreover, two distinct types of behavior can be identified as intergroup solidarity components: support for collective action and helping behaviors. Support for collective action is a joint effort, through which members of an outgroup collaborate with disadvantaged groups in addressing structural inequality problems (Radke, Hornsey & Barlow 2018). It may enhance disadvantaged group members’ empowerment, sense of independence, and self-advocacy (Nadler 2002). Thus, it is preferred by them compared to helping behaviors. Helping behaviors involve alleviating the suffering of others (Louis et al. 2019) with the aim to offer instant solutions to a specific issue and target, leaving untouched the structural system of inequalities. Yet, helping behaviors have a tendency to lean the symptoms, while not challenging the cause (Subašić, Reynolds & Turner 2008). Nevertheless, helping behaviors can provide essential assistance in circumstances where collective action takes more time and effort to obtain its results. Thus, these two complementary dimensions of solidarity tackle the complexity of the phenomenon, addressing different forms of intergroup cooperation. These two forms of solidarity differ from prosociality as they tackle the specifically oriented motivation to engage in challenging current inequalities against a group or a series of disadvantaged groups, rather than a general-individual tendency to be kind or altruist. Notwithstanding the differences in terms of predictors, dynamics, and outcomes between solidarity collective action and solidarity helping (Van Zomeren et al. 2008), both of them represent crucial options of engagement in activities that overcome indifference and status and represent a significant step in the direction of societal improvements. The majority of research focuses on majority group members’ solidarity toward ingroup members or a minority group (Kruse 2023), exclusively support for pro-(in/out)group policies (Cernat 2019; Visintin et al. 2017) is examined, leaving not yet fully addressed inter-minority solidarity. The present research aims to fill this gap by investigating the associations between inter-minority and majority-minority contact valence and solidarity between ethnic minorities, as cooperation for both instant helping and social change. Specifically, the present research aims to answer the following research questions across two distinct contexts: (a) Are (both positive and negative) direct contact experiences with other disadvantaged groups linked to ethnic minority members’ willingness to engage in solidarity with them? (b) Do negative emotions related to perceived injustice toward other disadvantaged groups contribute to explain these associations? (c) Can both positive and negative contact experiences with the majority group influence these associations?

## The Present Research

The present research aims to address the solidarity that ethnic minority group members pursue in contemporary societies. We aim to go beyond majority-minority relations (Dixon et al. 2020) by explicitly addressing the role of contact experiences of an ethnic minority with both other ethnic minorities and a majority group, in facilitating or inhibiting inter-minority solidarity (Brown & Ostrove 2013; Droogendyk et al. 2016; Louis et al. 2019). Not only positive contact among these groups but also its interplay with negative contact is examined, as prior work showed that negative contact is not merely the opposite of positive contact but entails qualitatively distinct antecedents, outcomes, and psychological mechanisms (Graf, Paolini & Rubin 2014; Schäfer et al. 2021). Moreover, both facets of intergroup solidarity, one related to support for collective action and the other related to intentional help in favor of other minority groups are assessed (see Table 1 for a summary of hypotheses). Specifically, we expect that positive inter-minority contact will be associated with solidarity intentions toward other ethnic minority groups to promote social equality (Hypothesis 1a). In contrast, negative inter-minority contact will be negatively associated with solidarity (Hypothesis 1b). Moreover, during contact with members of other ethnic minority groups, who share similar discrimination experiences (Sanchez, Kalkstein & Walton 2022), individuals should be more likely to express feelings of injustice toward shared conditions. Thus, we expect that positive inter-minority contact will be associated with affective injustice shared by both groups (Hypothesis 2a), whereas negative inter-minority contact will be negatively associated with affective injustice (Hypothesis 2b). Given that affective injustice is one of the main predictors of collective action for one’s group (van Zomeren et al. 2008), and more in general group-based emotions predict specific action tendencies (see Mackie et al. 2000), we expect that affective injustice for shared conditions will mediate the association between inter-minority contact and willingness to support another minority (i.e., solidarity), considering separately both forms of positive (Hypothesis 3a) and negative (Hypothesis 3b) contact. However, positive intergroup contact with the majority group attenuates ethnic minority members’ motivation to social change, because it is known to reduce perceived social inequalities and increase willingness to preserve positive relations with the majority group (Cernat 2019; Saguy et al. 2009). In this vein, we further explored the interplay between inter-minority and majority-minority contact valence (see Supplementary Materials, Table S1 for reasoning, hypotheses, and results).

**Table 1:** Summary of Studies 1 and 2 Hypotheses and Results.


	HYPOTHESES	STUDY 1 RESULTS (BELGIUM)	STUDY 2 RESULTS (TURKEY)

1 – The link between inter-minority contact and solidarity	1a – Positive inter-minority contact will be related to higher inter-minority solidarity	✓	✓

	1b – Negative inter-minority contact will be related to lower inter-minority solidarity		

2- The link between inter-minority contact and injustice	2a – Positive inter-minority experiences will be related to higher emotional shared injustice	✓	✓

	2b – Negative inter-minority experiences will be related to lower emotional shared injustice		×

3- The mediation of injustice	3a – Emotional shared injustice will mediate the link between positive inter-minority contact and inter-minority solidarity	✓	✓

	3b – Emotional shared injustice will mediate the link between negative inter-minority contact and inter-minority solidarity		×


Note. ✓ = results supported hypothesis; × = results showed opposite findings.

To test the hypotheses, two studies were conducted with understudied ethnic minority groups, North-African people living in Belgium (Study 1a), and Iraqi people living in Turkey (Study 1b). These minority groups are among the most represented and highly discriminated in the respective countries (Devos et al. 2024; Ihlamur-Öner 2013; Kislev 2018). In the last decades, these countries shared similar situations given that they have had to cope with a relatively large number of refugees, tapping resources, and forcing discussions and policy changes to accommodate the groups of people that do not speak their language and do not share most of their cultural practices (De Coninck et al. 2021). However, Turkey displays a lower socio-economic level, educational attainment, and weaker integration policies than Belgium (see MIPEX 2020), all factors contributing to increased hostility of native people toward migrants (De Coninck et al. 2021). Thus, we may expect a stronger role of both inter-minority and majority-minority contact experiences in the Turkish compared to the Belgian contexts, but overall, the studies were designed to assess the generalizability of findings across these different countries at the economic, educational and immigration policies levels.

## Cultural and Demographic Contexts of the studies

Study 1a investigates the link between intergroup contact and solidarity between ethnic minorities and with the majority group from the perspective of North-Africans living in Belgium (see Table 1). This is often overlooked as a country of immigration, even if over the last three decades Belgium has become a permanent country of settlement for many different types of migrants. In 2024, almost half of Belgians with a foreign background (46.4%) come from an European country, whereas the other half (53.6%) comes from a country outside Europe, and among them the highest percentage of migrants (27.2%) comes from North Africa (Statista 2024). Thus, North Africans are the biggest migrant group in the country with a different ethnic and cultural background from the host population and that does not share similar civic advantages granted to those coming from another European country (De Coninck et al. 2021). Specifically, Study 1a data collection took place in Brussels where approximately 32% of residents are of non-Belgian or non-European origin and 36% are second generation having different backgrounds, mostly Moroccan and Turkish (World Population Review 2024). This demographic information of Brussels (together with the information delivered by participants to the student who collected the data) indicates that Northern-African respondents thought with a high likelihood of Turkish immigrants and other Northern African immigrants in answering to the contact and solidarity questions toward other ethnic minority groups.

Study 1b investigates the same hypotheses of Study 1a with another ethnic minority group living in a different country characterized by a lower integration rate and immigration policy index (MIPEX 2020) than Belgium, namely Turkey (see Table 1). Turkey’s geopolitical position—bordering conflict-prone countries such as Syria and Iraq—has positioned the country as the world’s largest host of refugees over the past decade, due to persistent regional turmoil. As of 2023, over 3.6 million Syrians live in Turkey under temporary protection, followed by significant numbers of Iraqi, Afghan, and Iranian asylum seekers and migrants. Despite their long-standing presence and substantial population in Turkey, Iraqi refugees have increasingly been overshadowed by the mass arrival of Syrians since 2011 (Aşkar & Erdoğan 2023). This shift has reshaped public discourse on migration, redefined the concept of refugeehood in Turkey, and influenced both policy and the distribution of humanitarian aid—often to the disadvantage of other refugee groups, including Iraqis (Aşkar & Erdoğan 2023). Specifically, Study 1b data collection was conducted in collaboration with an NGO supporting Iraqi refugees in Ankara. Syrians are the largest ‘foreign’ population in Turkey, accounting for nearly 3.6 million people at the time of data collection, representing approximately 80% of the total foreign population. They have strong cultural and historical ties to Iraqi people. Both Iraqi and Syrian communities have faced displacement due to conflict and war (Aşkar & Erdoğan 2023). This demographic context and the third co-author’s short interviews conducted during data collection indicate that Syrians are very likely the ethnic minority that Iraqi participants had in mind when answering questions about inter-minority contact and solidarity.

Despite the contextual, cultural, and political differences characterizing the two countries and respective migrant groups, both of them (as other ethnic minorities) share: similar difficulties of adaptation in a host country that differ from their home country in terms of culture, tradition, religion, language; similar structural inequalities reserved to migrants compared to native people (i.e., housing, jobs) and similar personal injustices suffered in terms of micro-aggressions against them or their fellow ingroup members.

## Method

### Respondents and Procedure

***Study 1a.*** The sample comprised 301 first- and second-generation North-African students living in Belgium (*N* = 258 women; *M*_age_ = 21.86 years; *SD* = 5.57). On average, respondents came from the Maghreb region, specifically from three North-African countries (56.6% Morocco, 10.3% Tunisia, 3.3% Algeria) or having Maghrebi origins (29.6% second-generation migrants). Among the first-generation migrant students, respondents had been living in Belgium at least for two years (*M* = 15.78 years; *SD* = 13.63). Regarding respondents’ socio-economic status, 6% perceived their economic situation as better than most, 18.3% as good, 48,5% as the same as average people in the country, 8.6% as worse than most, 2% as poor, and 50 respondents did not reply to this question. The education level of the sample was as follows: no one stopped studying after receiving their elementary school diploma, 12% obtained a high school diploma, 84.1% had a university title (and 9 respondents did not reply). In terms of political orientation, 24.6% of respondents reported to be left-wingers, 22.3% were center-left supporters, 15.3% were center supporters, 0.7% were center-right supporters, 2% were right-wingers, 18.3% answered ‘other’, and 16.9% did not respond. Given that 65 respondents completed only the demographics at the beginning of the questionnaire, our final sample was composed of 236 North-African young adults (see Supplementary Materials, Table S2).

Collection of data was done through a self-report questionnaire available online, using Qualtrics software. All respondents were recruited via billposting at the University of Bruxelles and posting on social media. The questionnaire was formulated in English and then translated and back translated from French to suit the linguistic competence of each respondent. Respondents chose the language in which they completed the questionnaire. The study was previously approved by the University of [BLINDED FOR REVIEW] Ethics Research Committee. Datasets can be found at the following link: https://osf.io/hfxwq/.

***Study 1b.*** The sample comprised 210 first-generation Iraqi adults living in Turkey (*N* = 110 women; *M*_age_ = 34.54 years; *SD* = 13.09). Respondents had been living in Turkey for some years (*M* = 8.57 years; *SD* = 4.50). Regarding respondents’ socio-economic status, 9.6% perceived their economic situation as better than most, 11.9% as good, 33.8% as the same as average people in the country, 28.6% as worse than most, and 16.2% as poor. The education level of the sample was as follows: elementary school diploma (17.6%), high school diploma (55%), university title (25.2%), and 4 respondents did not respond. In terms of political orientation, the majority of respondents did not reply (58.6%), some were left-wingers (0.5%), center-left (0.5%), center (4.3%), center-right (1.0%), and right-wing (1.9%). Many of them reported ‘other’ (33.3%).

All respondents were legal migrants who attended multicultural activity centers in the capital city of the Republic of Turkey, Ankara. They were asked to complete a paper-pencil questionnaire on a voluntary basis. The questionnaire was formulated in English and then translated and back translated from Turkish and Arabic to match the linguistic competence of each respondent. Respondents chose the language in which they completed the questionnaire, and the third author answered any questions or doubts raised by them. This way, all participants who started to fill in the questionnaire completed the entire survey. The study was previously approved by the University of Bologna Ethics Research Committee.

### Measures

The following measures were used in both studies adapting to the specific contexts and groups:

**Positive and Negative Inter-Minority Contact** were assessed using one item each, with a response scale ranging from 1 (*never*) to 5 (*very often*): ‘How often do you have positive/negative experiences with people belonging to the main other ethnic minorities living in this country?’.

**Positive and Negative Contact With the Majority Group** were assessed using one item each, with a response scale ranging from 1 (*never*) to 5 (*very often*): ‘How often do you have positive/negative experiences with native [Belgians/Turkish people]?’.

**Affective Injustice** was assessed with four items (i.e., ‘I think the way ethnic minorities are treated by native people of this country is unfair’, ‘I feel angry because the work conditions of native people of this country are more advantageous than those of ethnic minority members’, ‘I am satisfied with the possibilities that are given to ethnic minorities living in this country.’, ‘I feel resentment about the disadvantaged life conditions of ethnic minority members compared to those of native people of the country’), with a scale ranging from 1 (*not at all*) to 5 (*very much*) forming reliable indexes, McDonald’s omegas = .70 (Study 1a); 71 (Study 1b).

**Inter-Minority Solidarity** was assessed using two items, with the response scale ranging from 1 (*never*) to 5 (*very often*): ‘To what extent would you be willing to cooperate with the other ethnic minorities to improve their life conditions in this country’ and ‘To what extent would you be willing to participate in demonstrations against injustices toward other ethnic minorities in this country’. The items in both studies were correlated, *r*(215) = .61, *p* < .001 (Study1a), *r*(208) = .64, *p* < .001 (Study 1b).

Other measures included in the questionnaires were not examined in this study.

## Results and Discussion

### Preliminary Analyses

Using GPower (Erdfelder, Faul & Buchner 1996), we conducted a sensitivity analysis for linear multiple regression, whereby the minimum effect size that indicates Study 1a is sufficiently powered, given the sample size (N = 236), is η^2^ = .04. In Study 1, the effect size of the interaction is higher than this number, indicating that the study is sufficiently powered. The same applied to Study 1b, with the sample size (N = 210) showing an interaction effect size higher than η^2^ = .05.

### Main Effects and Mediation Analyses

***Study 1a.*** Means, standard deviations and bivariate correlations are reported in Table 2. Positive inter-minority contact of North-Africans was associated with contact with the majority group of Belgian natives, both positive and negative experiences with the majority, indicating a general tendency to have interactions with members of different groups. Positive inter-minority contact was also associated with affective injustice of North-Africans and solidarity with other ethnic minority groups. Negative inter-minority contact instead was associated with negative contact with the majority group. Furthermore, positive and negative contact of North-Africans with the majority group in Belgium was associated with injustice toward other minority members. Moreover, affective injustice was significantly associated with inter-minority solidarity.

**Table 2:** Means, standard deviations and correlations among Study 1 variables.


	*M*	*SD*	2.	3.	4.	5.	6.

1. Positive inter-minority contact	3.88	0.95	0.08	0.29**	0.21**	0.19**	0.32**

2. Negative inter-minority contact	2.35	0.76		0.00	0.27**	0.11	0.10

3. Positive contact with the majority	3.22	1.08			0.12	0.16*	–0.03

4. Negative contact with the majority	2.41	0.81				0.14*	0.13

5. Emotional injustice	3.05	0.84					0.24**

6. Inter-minority solidarity	3.33	1.15					


*Note*. * *p* < .05, ** *p* < .01.

Process macro model 4 (Hayes 2022) was used to test the associations between inter-minority contact and solidarity and the mediating role of affective injustice, considering first positive inter-minority contact and second negative inter-minority contact (see Table 3). Results of the first regression model showed positive associations between positive inter-minority contact and solidarity (supporting Hypothesis 1a) and also between positive contact and affective injustice (supporting Hypothesis 2a). As expected, there was also a significant indirect effect of affective injustice (supporting Hypothesis 3a). Results of the second regression model showed no association between negative inter-minority contact and solidarity, nor with the mediator, thus no mediation effect of affective injustice (not supporting Hypotheses 1b, 2b, 3b).^1^

**Table 3:** Results for Estimated Coefficients of the Mediation Models (Study 1).


DV: INTER-MINORITY SOLIDARITY	IV: POSITIVE INTER-MINORITY CONTACT					IV: NEGATIVE INTER-MINORITY CONTACT				
	
	B	*SE B*	*T*	*R^2^*	*F*	B	*SE B*	*T*	*R^2^*	*F*

Constant	1.64	0.76	2.17**	0.13	8.24***	2.95	0.72	4.05***	0.01	0.65

IV	0.48	0.09	4.95***			0.17	0.12	1.39		

Socio-economic status	–0.05	0.09	–0.55			0.01	0.10	0.09		

Political orientation	–0.01	0.18	–0.03			–0.02	0.19	–0.11		

Constant	0.95	0.77	1.23	0.17	8.94***	1.90	0.75	2.52**	0.09	3.96**

IV	0.40	0.09	4.17***			0.12	0.11	1.05		

Emotional injustice	0.23	0.10	3.12**			0.38	0.10	3.71***		

Socio-economic status	–0.08	0.09	–0.91			–0.04	0.09	–0.47		

Political orientation	0.04	0.18	0.20			0.05	0.18	0.25		


*Note*. * *p* < .05. ** *p* < .01. *** *p* < .001.

***Study 1b.*** Means, standard deviations and bivariate correlations are reported in Table 4. Positive and negative inter-minority contact of Iraqi migrants with other ethnic minority groups were associated with positive and negative contact with the majority group, indicating a general tendency to have experiences with members of different groups. Positive and negative inter-minority contact were associated with affective injustice of Iraqi migrants and their motivation to work in solidarity with other ethnic minority groups. Negative contact with the majority group was positively associated with injustice. Moreover, there was a significant association between affective injustice and solidarity.

**Table 4:** Means, standard deviations and correlations among Study 2 variables.


	*M*	*SD*	2.	3.	4.	5.	6.

1. Positive inter-minority contact	2.16	1.02	0.65**	0.29**	0.16*	0.20**	0.32**

2. Negative inter-minority contact	2.05	0.98		0.17*	0.16*	0.28**	0.27**

3. Positive contact with the majority	2.86	0.93			0.09	0.03	–0.05

4. Negative contact with the majority	2.24	0.86				0.34**	0.09

5. Perceived injustice	2.08	0.82					0.28**

6. Inter-minority solidarity	2.14	1.10					


*Note*. * *p* < .05, ** *p* < .01.

As in Study 1a, regression models to assess the mediating role of affective injustice were conducted (see Table 5). In the first model, results showed positive associations of positive inter-minority contact with solidarity as well as injustice (supporting Hypotheses 1a and 2a). Moreover, an indirect effect of injustice, *b* = 0.04, *SE* = 0.02, 95% CI [0.01, 0.09] was found (supporting Hypothesis 3a). Results of the second model showed no significant association between negative inter-minority contact and solidarity and a positive association between negative inter-minority contact and the affective injustice, *b* = 0.18, *SE* = 0.04, 95% CI [0.10, 0.27] (not supporting Hypotheses 2a and 2b). Evidence also showed a partial mediation of affective injustice, but contrary to our expectations (Hypothesis 3b), affective injustice was positively (instead of negatively) associated with negative inter-minority contact. Overall, results across the two samples were consistent and partially supported our hypotheses, showing that positive (but not negative) inter-minority contact was linked with Northern Africans and Iraqi migrants’ intentions to work in solidarity with other ethnic minority groups living in their host country. This association was also consistently explained by affective injustice.

**Table 5:** Results for Estimated Coefficients of the Mediation Models (Study 2).


DV: INTER-MINORITY SOLIDARITY	IV: POSITIVE INTER-MINORITY CONTACT					IV: NEGATIVE INTER-MINORITY CONTACT				
	
	B	*SEB*	*T*	*R^2^*	*F*	B	*SEB*	*T*	*R^2^*	*F*

Constant	1.27	0.37	3.37***	0.11	8.67***	1.48	0.38	3.90***	0.07	5.60**

IV	0.38	0.07	5.09***			0.31	0.07	4.10***		

Socio-economic status	0.04	0.06	0.70			0.01	0.06	0.26		

Political orientation	–0.05	0.07	–0.80			–0.03	0.07	–0.44		

Constant	0.89	0.39	2.25**	0.15	8.81***	1.15	0.39	2.90**	0.10	5.96***

IV	0.34	0.07	4.57***			0.26	0.07	3.32**		

Emotional injustice	0.26	0.09	2.88**			0.24	0.09	2.56**		

Socio-economic status	0.02	0.06	0.42			0.00	0.06	0.03		

Political orientation	–0.05	0.07	–0.82			–0.03	0.07	–0.45		


*Note*. * *p* < .05. ** *p* < .01. *** *p* < .001.

### General Discussion

In this contribution we addressed the interplay between inter-minority and majority-minority contact and their relations with affective injustice and willingness to work in solidarity.

Convergent results were found across the two studies investigating highly discriminated ethnic minorities in contexts characterized by different levels of integration. Specifically, having positive inter-minority contact was linked with willingness of solidarity with other ethnic groups (supporting Hypothesis 1a). Moreover, positive inter-minority contact was linked with affective injustice (supporting Hypothesis 2a) which in turn mediated the association between inter-minority contact and solidarity (supporting Hypothesis 3a) consistently across studies. In contrast, negative inter-minority contact was neither significantly associated with solidarity nor with affective injustice, showing no mediation in Study 1a, whereas it was positively associated with solidarity, and affective injustice partially mediated this unexpected association in Study 1b (not supporting Hypotheses 1b, 2b, 3b). These inconsistent findings across the two studies suggest that the quality of inter-minority experiences has different implications depending on the context. In countries characterized by less anti-discrimination policies toward immigrants, ethnic minority members may perceive that contact with other ethnic groups, independently of its quality, provides a connection to build a potential reciprocal support to improve their conditions. Instead, in contexts characterized by higher support toward immigrants, the quality of inter-minority contact may play a stronger role in shaping their willingness to cooperate for the common goal of improving their life conditions.

Although not hypothesized, the consistent correlation between positive inter-minority contact and positive contact with the majority across both samples is noteworthy. It may rely on the need of minority members to establish good relations with different societal partners that have different roles and functions in facilitating ethnic minority groups’ adaptation in the host society. Positive contacts with majority members can contribute to feelings of being accepted in the host country, whereas positive inter-minority contact can serve the function of driving ethnic minority group members to unify their effort in striving to achieve better conditions through collective action. Similarly, in both studies results showed a positive correlation between negative contact with the majority group and negative contact with other minority groups. This evidence highlights a general tendency to perceive contact with other group members as difficult, threatening or conflictual, no matter the type of group considered. Ethnic minority members belonging to a disadvantaged group can feel marginalized in many different aspects (i.e., cultural, economic, political) and thus have difficulties in building connections not only with the majority group but in general with outgroup members who may be perceived as not sharing similar disadvantages. Finally, in line with the literature and previous research (Schäfer et al., 2021 for a review), no significant association was found between positive and negative types of (majority-minority and minority-minority) contact, except for inter-minority contact in the Turkish sample. In this case, having positive inter-minority contact was significantly associated with having negative ones.

### Theoretical and Practical Implications

The present cross-sectional studies offer theoretical insights into the understudied dynamics of inter-minority solidarity (Dixon et al. 2016). Results extend our understanding in several ways, answering also to the need to move beyond attitudinal outcomes, to address specific behavioral intentions of solidarity between groups in the intergroup contact literature (Policardo, Karataş & Prati 2025). First, they consistently underscore the pivotal role of positive contact in fostering solidarity among ethnic minority outgroups. The collected evidence supports the existing literature on the benefits of intergroup contact (Pettigrew & Tropp 2006), by addressing its role among disadvantaged groups in promoting not only support for collective action of outgroups but also cooperative behavioral intentions among groups (Dixon et al. 2020). Thus, the present research supports and extends previous evidence (Cernat 2019; Visintin et al. 2017) by indicating positive inter-minority experiences as a factor linked to solidarity among groups. The consistent outcomes across different groups and contexts suggests the generalizability of this path and its implications in promoting solidarity in more and less inclusive societies. Differently from our expectations, we did not find a negative association between negative inter-minority contact and solidarity across any group. This result, on one side, differentiates dynamics among minorities from majority-minority ones, whereby negative experiences are linked to endorse minority groups’ collective action intentions (Reimer & Sengupta 2023). On the other side, it suggests that encouraging inter-minority experiences contributes overall to build cooperation among disadvantaged groups to change the system. Indeed, contrary to the contact valence asymmetry hypothesis suggesting that negative contact is stronger than positive contact in shaping intergroup attitudes (Barlow et al. 2012), our findings indicate a stronger role of positive inter-minority contact in engaging in actions to reduce social inequalities.

Moreover, the present findings contribute to the literature of intergroup contact and collective action, by addressing the role of affective injustice to explain the link between these two factors. The evidence implies that positive encounters among members of ethnic minorities are perceived as a good opportunity to share reciprocal feelings and awareness of experienced injustices. In this vein, positive inter-minority contact per se represents a way to build support and solidarity among groups members that suffer and are persistently targets of discrimination. While this research successfully identified the mediating role of injustice across both countries (Belgium and Turkey), it leaves room to explore additional factors that may explain the relationship between contact and solidarity. Future research can investigate the strength of other psychological processes underpinning this association, such as perceived minority group similarities or competitive victimhood (Ball & Branscombe 2019). Contrary to our expectations, evidence did not show any detrimental role of negative inter-minority contact on affective injustice and motivation to reciprocal support for social change. Unexpectedly, in the Turkish context, negative inter-minority contact was positively associated to ethnic minority members’ awareness of suffered common injustices. Notwithstanding the cross-country inconsistent results calling for more research and more specific measures of the quality of contact, this preliminary finding seems to highlight a stronger role of the frequency of inter-minority connections than their quality in fostering solidarity among them in less supportive contexts, such as Turkey compared to Belgium. Yet, not only contextual but also demographic differences across the two samples may explain this inconsistency, with Iraqi migrants being slightly older than Northern African migrants and thus experiencing not only different quality of negative intergroup contact, but also less personal status precariousness to facilitate inter-minority solidarity independently of the type of interaction. This consideration is also in line with the specific (and in contrast with the literature) finding in the Turkish context showing that positive and negative inter-minority contact were significantly correlated.

Beyond its theoretical contributions, this research also offers practical insights. Most contact-based interventions have historically concentrated on majority–minority relations; however, the present findings emphasize the importance of facilitating positive interaction between different minority groups in increasing multicultural societies. In this regard, interventions targeting inter-minority contact in schools, neighborhoods, and community organizations could play a key role in empowering marginalized or disadvantaged groups to become mutual allies in addressing structural inequalities.

### Limitations and Future Directions

While this research provides valuable insights into the dynamics of inter-minority contact and solidarity, it is important to acknowledge several limitations.

First of all, the cross-sectional design of the studies does not allow clearcut inferences about causality between the measured variables. Future studies should investigate underlying processes of inter-minority contact on solidarity using longitudinal designs. Moreover, the present hypotheses were not pre-registered. While the research was theoretically motivated and the analyses were planned prior to data collection, the absence of a formal pre-registration requires interpreting the findings with some caution and encourages future pre-registered research to enhance the transparency and replicability of these findings. Another limit is the use of single-item measures, particularly in assessing the frequency of positive and negative intergroup contact. While the single-item approach is a common method in social research, it may not capture the full complexity of contact experiences. Future studies could benefit from more comprehensive measures that encompass various dimensions of intergroup contact (Lolliot et al. 2015).

Moreover, the measures of contact and solidarity employed are referred to the main other ethnic minority groups in the host country. Future research could focus on solidarity with specific ethnic minority groups, assessing the role of groups status as in previous research (Cernat 2019). Indeed, while our results were rather consistent across different contexts, given that certain immigrant group’s characteristics (e.g., socio-economic status) could influence the link between contact and solidarity, caution should be taken when generalizing these findings to different immigrant groups in other settings. In addition, our measures of solidarity captured participants’ willingness to engage in action, rather than actual participation. While intentions are an important predictor of behavior (Ajzen 1991), given that recent research showed some difference between solidarity tendencies and real participation in solidarity action (Carmona et al. 2025), future studies should include behavioral indicators of inter-minority solidarity to assess whether and how these intentions translate into real-word engagement.

These limitations do not diminish the significance of the study’s findings but rather point to opportunities for future research to delve deeper into the complexities of the phenomenon.

## Conclusions

The two studies conducted on processes involved in inter-minority contact and solidarity offer valuable insights into the understudied dynamics of intergroup relations among ethnic minority groups. They invite to take into account the different forms of intergroup contact, their valence and their underlying processes in supporting collective effort intentions as potential precursor to real-word engagement directed toward building resilient and cohesive communities.

## Additional File

The additional file for this article can be found as follows:

10.5334/irsp.1096.s1 Supplementary Materials. Tables S1–S4 and Figures S1–S5.

## Footnotes

All main analyses of Study 1 were also conducted including immigration generation of participants (first or second) as further covariate and results did not change.
