# Supplementary material for: Fostering Solidarity Among Ethnic Minority Groups: Addressing the Role of Inter-Minority Contact in Cross-Cultural Contexts
Source: Int Rev Soc Psychol. 2025 Oct 13;38:14. doi: 10.5334/irsp.1096 (PMC12539338; doi:10.5334/irsp.1096)
Supplement: Supplementary Materials. — Tables S1–S4 and Figures S1–S5. [file irsp-38-1096-s1.pdf]

Supplementary Materials

	Hypotheses	Study 1 Results (Belgium)	Study 2 Results (Turkey)
4 & 5 – The interplay between inter-minority and majority-minority contact valence	4a - Positive inter-minority contact will decrease the link between positive contact with the majority and lower inter-minority solidarity	✓	✓
	5a - Positive inter-minority contact will facilitate the link between negative contact with the majority and higher inter-minority solidarity	✓	✓
	4b - Negative inter-minority contact will facilitate the link between positive contact with the majority and lower inter-minority solidarity		×
	5b - Negative inter-minority contact will decrease the link between negative contact with the majority and higher inter-minority solidarity		

Table S1. *Summary of Studies 1 and 2 Moderation Hypotheses and Results.*

Moderation Hypotheses

Going beyond previous research (Cernat et al., 2019), we expect the negative association between positive contact with the majority group and solidarity with other ethnic minorities will be attenuated by positive inter-minority contact (Hypothesis 4a) and enhanced by negative inter-minority contact (Hypothesis 5b). In contrast, considering that negative contact with the majority group should facilitate the motivation to endorse social change (Hässler et al., 2020), the association between negative contact with the majority and inter-minority solidarity should be increased by positive inter-minority contact (Hypothesis 5a) and reduced by negative inter-minority contact (Hypothesis 4b).

Table S2.

Attrition for Respondents (those who completed both all main variables vs. missing)

	Complete responses (N = 236)	Missing (N = 65)	Significant difference and Cohen's *d*
Age	22.05	21.20	0.17
Income	3.76	3.84	0.09
Political orientation	2.99	2.35	-0.35*
Education	3.85	3.92	0.21

Note. Little's MCAR test: Chi-Square = 4.140, DF = 3, p = .247.

In Study 1, we tested for systematic attrition between respondents who completed all the variables (N = 236) and those who only completed only the demographics part (N = 65). Results indicated that respondents who completed all measures were significantly more left-wing than those who did not.

Table S3.

Results for Estimated Coefficients of the Moderation Models (Study 1).

DV: Inter-minority solidarity	IV: Positive inter-minority contact					IV: Negative inter-minority contact				
	B	*SE B*	*t*	R^2	*F*	B	*SE B*	*t*	R^2	*F*
Constant	3.30	0.07	43.78***	0.14	11.54***	3.35	0.07	43.07***	0.01	0.76
IV	0.47	0.08	5.80***			0.14	0.10	1.43		
Positive contact with the majority	-0.13	0.07	-1.82			0.02	0.07	0.25		
IV × positive contact with the majority	0.12	0.06	2.11*			0.03	0.08	0.33		
Constant	3.29	0.07	43.57***	0.12	9.07***	3.33	0.08	41.68***	0.02	1.67
IV	0.40	0.08	4.80***			0.10	0.10	0.95		
Negative contact with the majority	0.10	0.09	1.09			0.15	0.09	1.55		
IV × negative contact with the majority	0.17	0.08	1.93*			0.09	0.12	0.73		

Note. * $p < .05$. ** $p < .01$. *** $p < .001$.

Table S4.

Results for Estimated Coefficients of the Moderation Models (Study 2).

DV: Inter-minority solidarity	IV: Positive inter-minority contact					IV: Negative inter-minority contact				
	B	*SE B*	*t*	R^2	*F*	B	*SE B*	*t*	R^2	*F*
Constant	2.09	0.07	28.33***	0.14	11.67***	2.09	0.07	28.99***	0.13	10.17***
IV	0.37	0.07	5.13***			0.30	0.07	4.06***		
Positive contact with the majority	-0.17	0.07	-2.16**			-0.12	0.07	-1.54		
IV × positive contact with the majority	0.15	0.07	2.05*			0.23	0.07	3.32***		
Constant	2.10	0.07	29.12***	0.13	10.33***	2.10	0.07	28.18***	0.08	1.11***
IV	0.33	0.07	4.71***			0.28	0.07	3.75***		
Negative contact with the majority	0.03	0.08	0.38			0.06	0.08	0.68		
IV × negative contact with the majority	0.16	0.07	2.37**			0.14	0.09	1.53		

Note. * $p < .05$. ** $p < .01$. *** $p < .001$.

Moderation Analyses and Results

In both studies, Process macro model 1 (Hayes, 2022) was used to test the moderating roles of positive and negative inter-minority contact separately first on the association between positive contact with the majority group and inter-minority solidarity and second on the association between negative contact with the majority group and inter-minority solidarity (see Tables S3 and S4).

Study 1a. In the first regression model, results showed an interaction between positive inter-minority contact and positive contact with the majority group (supporting Hypothesis 4a). Tests of simple slopes (see Figure S1) showed that for North-Africans reporting relatively lower positive inter-minority contact, positive contact with the majority group was negatively associated with solidarity between minority groups, b = -0.24, SE = 0.08, 95 % CI [-0.40, -0.07]. The same association was not significant among North-Africans reporting higher positive inter-minority contact, b = 0.01, SE = 0.06, 95 % CI [-0.19, 0.21], pointing to a buffering effect of positive inter-minority experiences on the detrimental role of positive contact with the majority group on inter-minority solidarity. In the second regression analysis, results showed a slightly significant interaction between negative contact with the majority group and positive inter-minority contact (supporting Hypothesis 5a). Tests of simple slopes showed that for North-Africans reporting relatively higher positive inter-minority contact, negative contact with the majority group was positively associated with solidarity between minority groups, b = 0.29, SE = 0.14, 95 % CI [0.02, 0.56]. The same association was not significant among North-Africans reporting lower positive inter-minority contact, b = -0.04, SE = 0.12, 95 % CI [-0.27, 0.18], pointing to a facilitating effect of positive inter-minority experiences on the association between negative contact with the majority group and inter-minority solidarity (see Figure S2). Two regression models further tested the moderating role of negative inter-minority contact, showing no significant interaction with positive contact with the majority group nor negative contact with the majority group (not supporting Hypotheses 4b and 5b).

Study 1b. Moderation models were tested to assess the roles of first positive inter-minority contact and then negative inter-minority contact on the associations between both positive and negative contact with the majority and inter-minority solidarity (see Table S4). In the first model, there was an interaction between positive inter-minority contact and positive contact with the majority group (supporting Hypothesis 4a). As shown in Figure S3, tests of simple slopes across scores of positive inter-minority contact showed a stronger significant negative association between positive contact with the majority and inter-minority solidarity for Iraqi migrants reporting relatively lower positive inter-minority contact, b = -0.35, SE = 0.11, 95 % CI [-0.58, -0.13], whereas this association was no longer significant for Iraqi migrants with high positive inter-minority contact, b = -0.03, SE = 0.11, 95 % CI [-0.25, 0.17]. As for Study 1a, in the second model, there was an interaction also between negative contact with the majority group and positive inter-minority contact (supporting Hypothesis 5a). As shown in Figure S4, tests of simple slopes across scores of positive inter-minority contact showed no significant association between negative contact with the majority group and solidarity with minorities for Iraqi migrants reporting relatively lower positive inter-minority contact, b = -0.16, SE = 0.12, 95 % CI [-0.41, 0.08]. However, the association between negative contact with the majority group and solidarity with other minorities for Iraqi migrants reporting higher positive inter-minority contact was closed to be significant, b = 0.17, SE = 0.09, 95 % CI [-0.00, 0.36]. Differently from Study 1a, a significant interaction between negative inter-minority contact and positive contact with the majority group was found (see Figure S5). Tests of simple slopes across scores of negative inter-minority contact showed no significant association between positive contact with the majority group and solidarity with minorities for Iraqi migrants reporting relatively higher negative inter-minority contact, b = 0.10, SE = 0.10, 95 % CI [-0.10, 0.31]. However, there was a significant negative association between positive contact with the majority group and solidarity with other minorities for Iraqi migrants reporting lower negative inter-minority contact, b = -0.36, SE = 0.10, 95 % CI [-0.57, -0.16]. Contrary to our expectations, having positive connections with the majority group was associated with lower support for other minorities

especially for Iraqi migrants with lower rather than higher negative inter-minority contact (not supporting Hypothesis 4b). Instead, no significant interaction between negative inter-minority contact and negative contact with the majority group (not supporting Hypothesis 5b). Overall, results of the moderations were consistent across the two samples, showing that having positive inter-minority contact attenuated the sedative role of positive contact with the majority on inter-minority solidarity, whereas it enhanced the association between negative majority-minority contact and inter-minority solidarity. Moreover, only Study 1b, contrary to our expectations, having negative inter-minority contact attenuated the negative association between positive contact with the majority group and support for other minorities.

Moderation Discussion

Going beyond previous findings on the interplay between the quality of majority-minority and inter-minority contact (Cernat, 2019), we found that across the two studies, positive inter-minority contact moderated the associations between both types of contact with the majority group and inter-minority solidarity. Having higher compared to lower positive inter-minority contact attenuated the detrimental role of positive contact with the majority group on solidarity with other minorities (supporting Hypotheses 4a) and enhanced the link between negative contact with the majority group and inter-minority solidarity (supporting Hypothesis 5a). No moderation of negative inter-minority contact was found except for the Turkish context, where having higher negative inter-minority contact reduced the negative association between positive contact with the majority group and solidarity (not supporting Hypothesis 4b).

Furthermore, our evidence emphasizes the benefits of inter-minority contact, by showing its buffering effect in attenuating the association between positive contact with the majority group and reduced solidarity among ethnic minority groups. These results, using a general measure of positive contact rather than the friendship measure used by Cernat (2019), were consistent across studies and highlight the role of inter-minority experience as a potential protective condition that acts as a safeguard against the paradoxical effects of positive contact with the majority in inhibiting

minorities’ search for justice. Results also supported the beneficial role of positive inter-minority contact in facilitating the link between negative contact with the majority and solidarity with other minority groups. Overall, evidence highlights the inter-twined role of contact with majority and other minority groups as two distinct actors to build connections with and work for social change in favour of ethnic minority members. Given the significant associations between different valenced contact and intergroup attitudes, future research might address the interplay between majority-minority and inter-minority contact by considering not only positive and negative experiences but also ambiguous ones to understand how they may come into play in addressing social change. Especially in the relations with outgroup members, individuals may experience some misunderstandings and difficulties that can be perceived differently also based on individual characteristics. Thus, further research may take into account ambiguous experiences broadening our understanding of the complex inter-play of contact with higher and lower status outgroups.

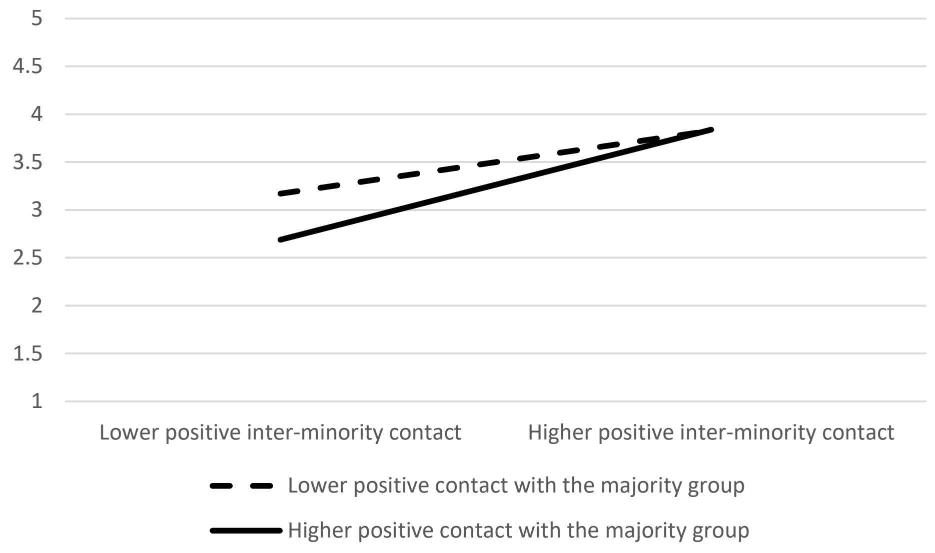

Figure S1. *North-African respondents' solidarity with other ethnic minority groups as a function of positive inter-minority contact and positive contact with the majority (Study 1).*

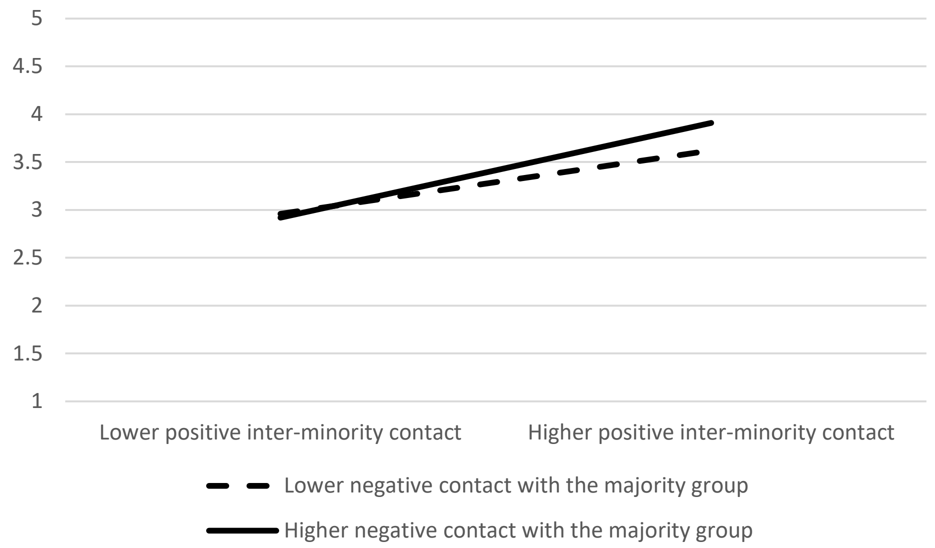

Figure S2. *North-African respondents' solidarity with other ethnic minority groups as a function of positive inter-minority contact and negative contact with the majority (Study 1).*

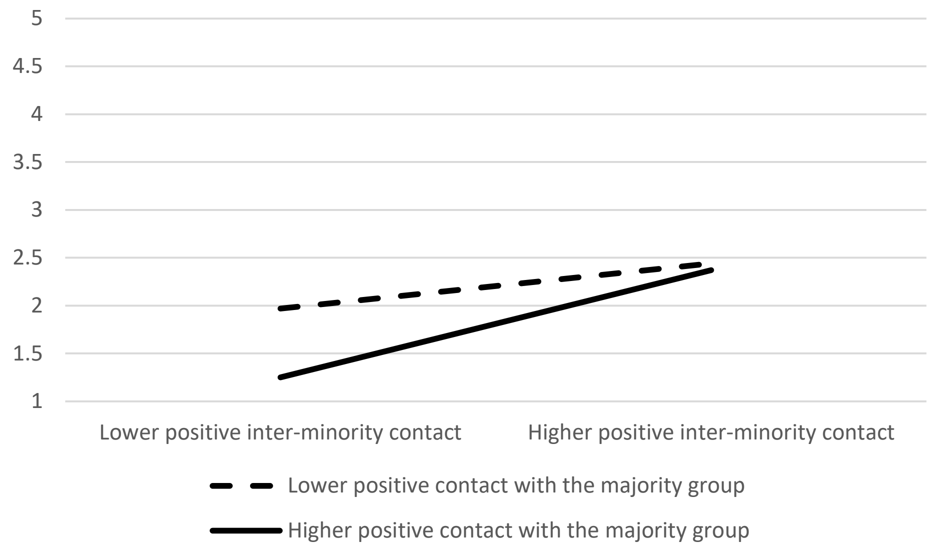

Figure S3. *Iraqi respondents' solidarity intentions toward other ethnic minority groups as a function of positive inter-minority contact and positive contact with the majority (Study 2).*

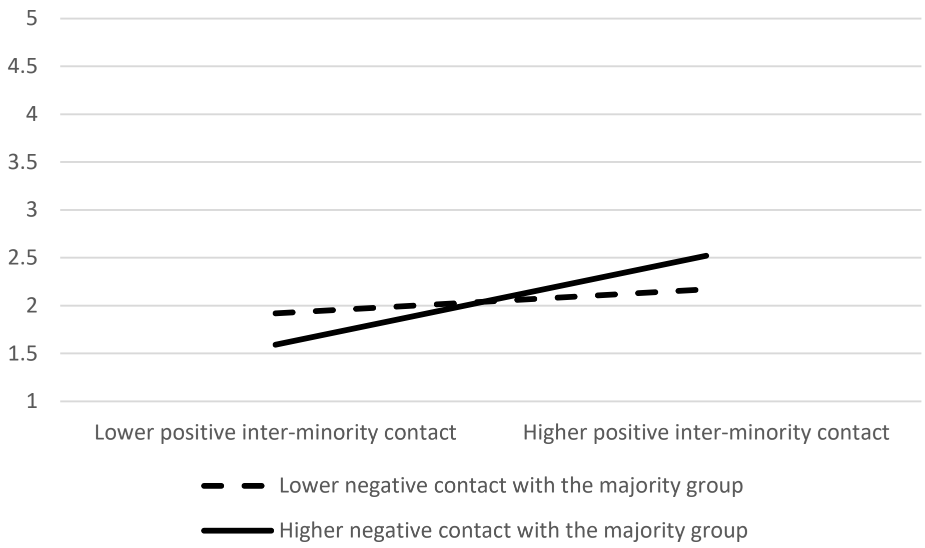

Figure S4. *Iraqi respondents' solidarity intentions with other ethnic minority groups as a function of positive inter-minority contact and negative contact with the majority (Study 2).*

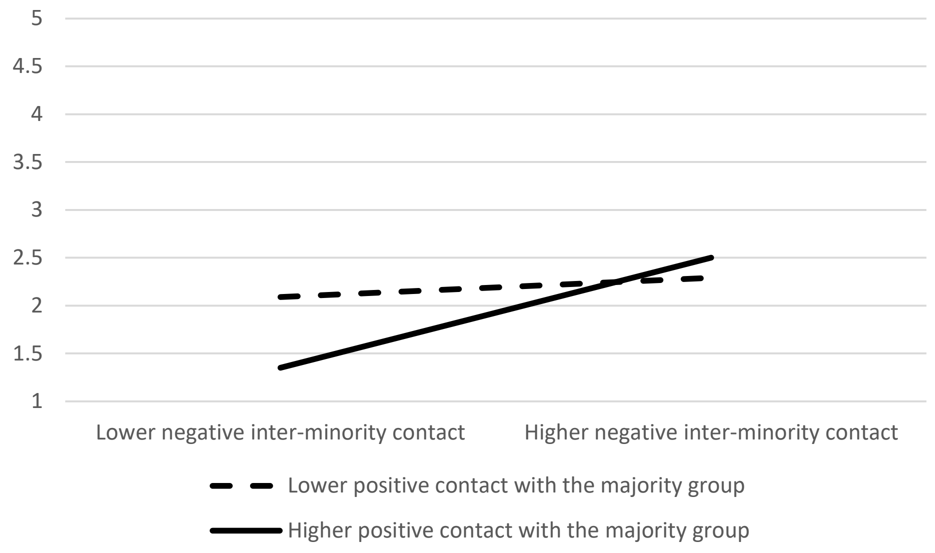

Figure S5. *Iraqi respondents' solidarity intentions with other ethnic minority groups as a function of negative inter-minority contact and positive contact with the majority (Study 2).*
